# MAFF is a transcription factor that regulates contraction-induced skeletal muscle transcription

**DOI:** 10.26508/lsa.202603709

**Published:** 2026-09-08

**Authors:** Taku Fukushima, Taiju Fujioka, Satoru Masubuchi, Iori Sakakibara

**Affiliations:** 1 Department of Physiology, School of Medicine, Aichi Medical University , Nagakute, Japan; 2 Laboratory of Stem Cell Regeneration and Adaptation, Graduate School of Pharmaceutical Sciences, The University of Osaka , Suita, Japan

## Abstract

Skeletal muscle adapts to exercise through rapid transcriptional remodeling, but regulators that link contractile activity to these gene programs remain unclear. Here we show that MAFF, a small MAF transcription factor, is consistently induced by acute exercise in both human and mouse skeletal muscle, with induction restricted to muscles recruited by the exercise modality. In C2C12 cells, *Maff* was induced by in vitro electrical pulse stimulation model. Using CRISPR/Cas9–mediated *Maff* knockout C2C12 cells, we found that loss of *Maff* reduced myogenic fusion with accumulation of unfused nuclei. RNA sequencing revealed broad reprogramming in *Maff*-deficient myotubes, including reduced expression of muscle structural and contractile genes and increased interferon/immune responses and extracellular matrix–related signatures. Moreover, *Maff* deficiency attenuated a subset of electrical pulse stimulation–responsive genes. Together, these data identify MAFF as a component of the transcriptional machinery supporting myogenic maturation and contraction-evoked gene regulation.

## Abstract (precis)

Acute exercise robustly induces MAFF in human and mouse skeletal muscle, and loss of *Maff* in C2C12 myotubes impairs myogenic fusion and attenuates contraction-induced transcriptional programs.

## Introduction

Skeletal muscle adapts to mechanical and metabolic demands through extensive transcriptional remodeling, which supports changes in metabolism, muscle fiber type composition, and functional capacity in response to exercise (Egan & Zierath, 2013; Hawley et al, 2014). Exercise-induced transcriptional responses vary depending on exercise modality, such as aerobic versus resistance exercise, as well as exercise frequency, including single-bout and repeated exercise paradigms (Pillon et al, 2020; Furrer et al, 2023).

Transcriptional responses to exercise are mediated by coordinated activities of multiple transcription factor families, including basic leucine zipper (bZIP) transcription factors such as FOS, JUN, and ATF3, which integrate mechanical, metabolic, and redox cues into gene expression programs (Puntschart et al, 1998; Popov et al, 2019). Members of the small MAF (sMAF) family (MAFF, MAFG, and MAFK) serve as bZIP partners for diverse transcriptional regulators and have been implicated in stress responses and differentiation processes in several cell types (Motohashi et al, 2004; Kannan et al, 2012; Katsuoka & Yamamoto, 2016).

Recent studies have identified large Maf transcription factors as key regulators of myosin heavy chain expression and fast-twitch muscle specification. In mice, the large MAF family (MAF, MAFA, and MAFB) was shown to specify fast type IIb fiber identity by directly driving *Myh4* expression, with knockout (KO) of large *Mafs* resulting in loss of type IIb fibers and altered muscle performance (Sadaki et al, 2023). In addition, large MAFs robustly induced *MYH4* expression and enhanced glycolytic metabolic capacity in human and bovine myotubes, and their expression correlated with fast-twitch myosin gene expression in human muscle biopsies, including samples from power-trained individuals (Sadaki et al, 2025). Consistent with a broader role for Maf factors in fast-muscle gene programs, single-nucleus analyses suggested that *Maf* acts as a transcriptional switch during myofiber maturation to activate mature fast gene expression and that impaired *Maf* expression accompanies defective maturation in a CaV1.1-deficient context (Dos Santos et al, 2023). Moreover, *Maf* has been linked to maintenance of fast-fiber gene expression in neuromuscular diseases, where *Maf* overexpression mitigated denervation-associated atrophy and restored fast gene expression in vivo and in human skeletal muscle cells (Dos Santos et al, 2025). These findings establish the large MAF family as key regulators of skeletal muscle transcriptional programs, particularly in fast-twitch fibers. However, the role of small Maf proteins, including MAFF, in exercise-responsive gene regulation in skeletal muscle remains incompletely understood.

sMaf proteins are broadly expressed across tissues, although their transcript abundance differs markedly among organs (Blank & Andrews, 1997). In several contexts, sMaf proteins form homodimers that are associated with transcriptional repression (Igarashi et al, 1994; Motohashi et al, 2000, 2006). By contrast, when sMaf proteins heterodimerize with Cap’n’Collar (CNC) family factors, these complexes have been described as transcriptional activators that promote target gene expression (Wild et al, 1999; Katsuoka & Yamamoto, 2016). Recent transcriptomic studies have suggested that *Maff* expression is induced by acute exercise in skeletal muscle, raising the possibility that MAFF contributes to exercise-responsive gene regulation (Pillon et al, 2020). In other tissues, MAFF expression has been shown to be inducible by proinflammatory cytokines, and MAFF has been implicated as a regulator of inflammation-linked metabolic gene networks in vivo. Moreover, the murine *Maff* locus has been molecularly characterized, including its promoter usage and tissue expression patterns (Onodera et al, 1999; Massrieh et al, 2006; von Scheidt et al, 2021). The *Maff* gene is composed of two coding exons, resembling the genomic organization of the related small Maf genes *MafG* and *MafK*. In addition, transcription can be initiated from multiple alternative promoters that are spliced to a shared second exon, resulting in several *Maff* transcripts that encode an identical protein product. However, whether MAFF plays a functional role in skeletal muscle adaptation to exercise remains unclear. In this study, we investigated the role of MAFF in skeletal muscle transcriptional responses using a combination of public exercise transcriptome datasets, in vivo exercise experiments, and in vitro electrical pulse stimulation (EPS) model in C2C12 myotubes (Fukushima et al, 2021). By integrating genetic ablation of *Maff* with transcriptome-wide analyses, we examined how MAFF influences contractile activity-dependent gene expression and myogenic transcriptional programs in skeletal muscle cells.

## Results

### Acute exercise and contractile stimulation induce *Maff* expression in skeletal muscle and C2C12 myotubes

**Figure 1.**
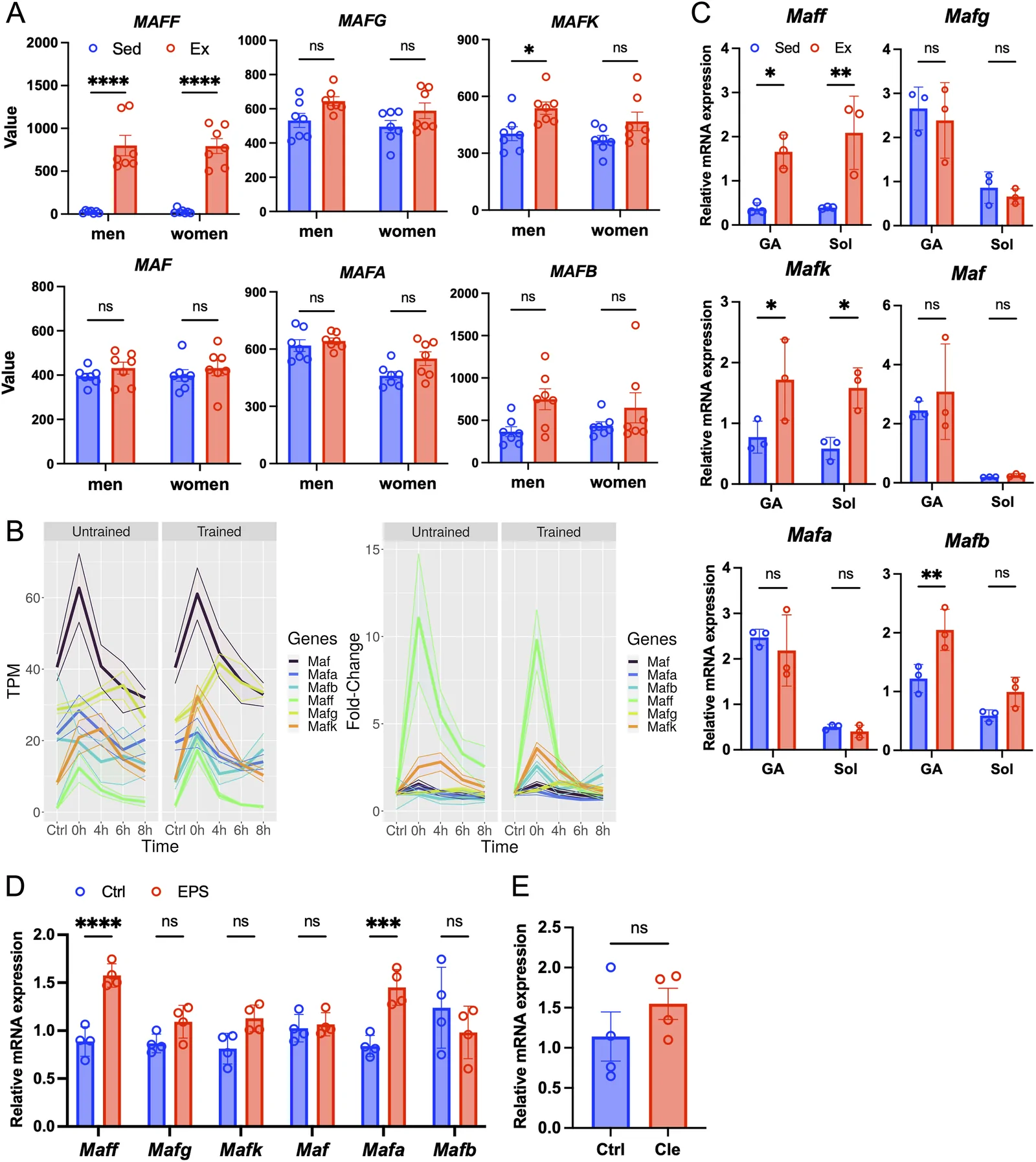
Exercise- and contractile stimulation-responsive expression of *Maff* in skeletal muscle and C2C12 cells. **(A**) Microarray analysis of *MAF* family gene expression in human sprint exercise (n = 7/group). **(B)** Time-course analysis of *Maf* family gene expression expressed as transcripts per million (TPM) and fold-change after acute exercise in mice (n = 5/group). **(C)** *Maf* family mRNA expression in the tibialis anterior (TA), gastrocnemius (GA), and soleus (Sol) muscles of mice after treadmill exercise (n = 3/group). **(D)** *Maf* family mRNA expression in C2C12 myotubes after electrical pulse stimulation (EPS) (n = 4/group). **(E)** *Maff* mRNA expression in C2C12 myotubes after clenbuterol treatment (n = 4/group). In (A, C, D), data are presented as mean ± SEM. *P < 0.05; **P < 0.01; ***P < 0.001; ****P < 0.0001; ns, not significant. Statistical significance was determined by two-way ANOVA or Welch’s *t* test, as indicated.

*Maff* has been reported to be induced by short-term exercise (Rundqvist et al, 2019; Pillon et al, 2020). To systematically evaluate the expression responsiveness of *MAF* family genes (*MAFF*, *MAFG*, *MAFK*, *MAF*, *MAFA*, and *MAFB*), we analyzed publicly available transcriptomic datasets obtained from acute exercise experiments in humans and mice (Rundqvist et al, 2019; Furrer et al, 2023). In a microarray dataset of human acute sprint exercise (GSE126296), *MAFF* expression was significantly increased after exercise, independent of sex (Fig 1A). In a mouse acute exercise dataset (Myo-TrEx, GSE221210), the large *Maf* transcription factor *Maf* exhibited higher basal expression levels when assessed as transcripts per million (TPM). In contrast, fold-change analysis revealed that *Maff* showed the greatest induction among the small MAF family members immediately after acute exercise, although *Mafk* expression was also moderately increased (Fig 1B). Consistent with these observations, analysis of MAF family gene expression in our in vivo exercise model showed that both *Maff* and *Mafk* were significantly induced in the gastrocnemius (GA) and soleus (Sol) muscles after exercise, whereas *Mafb* expression was increased only in the GA muscle (Fig 1C). These findings further support *Maff* as the most consistently exercise-responsive member of the MAF family.

We next evaluated *Maff* expression using in vitro exercise-mimicking system in differentiated C2C12 myotubes, including electrical pulse stimulation (EPS) and treatment with the β2-adrenergic receptor agonist clenbuterol (Cle) (Fukushima et al, 2021). EPS, but not Cle treatment, induced *Maff* expression in myotubes (Fig 1D and E). In contrast, *Mafk* expression was not significantly altered by EPS stimulation, whereas both *Maff* and *Mafa* were significantly induced (Fig 1D), suggesting that *Maff* is the most responsive small MAF family member to exercise-mimicking contractile stimulation. Together, these data demonstrate that *Maff* is acutely induced by exercise in both humans and mice and is responsive to exercise-mimicking contractile stimulation in C2C12 myotubes.

### Loss of *Maff* is associated with reduced myotube fusion in C2C12 cells

**Figure S1.**
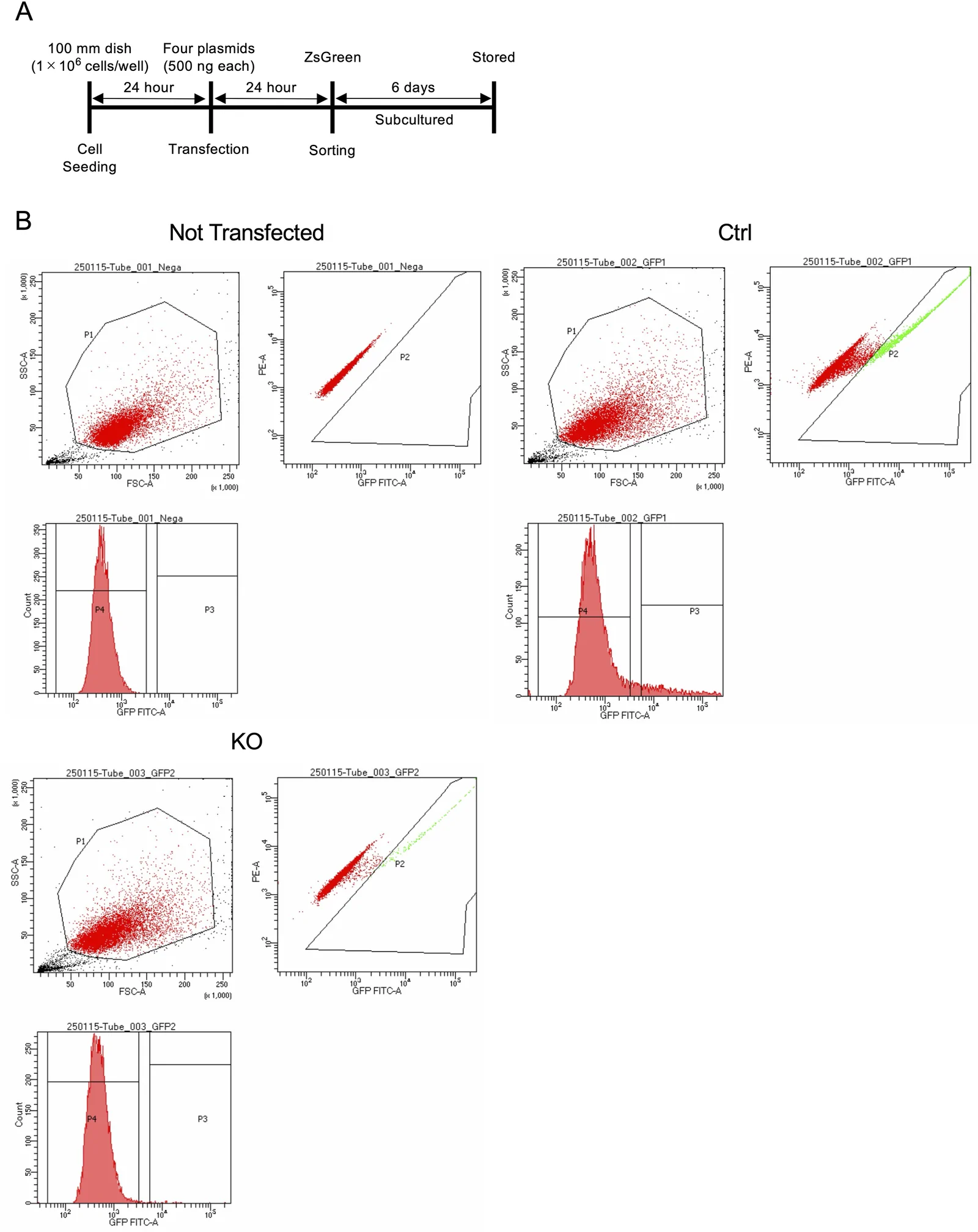
Generation of *Maff* KO C2C12 cells. **(A)** Schematic of the generation of *Maff* KO C2C12 cells. **(B)** ZsGreen-positive myoblasts were isolated by cell sorting. Untransfected C2C12 cells (no fluorescence) were used as negative controls. Ctrl and *Maff* KO cells were sorted under identical conditions. Sorted cells were indicated in the green areas.

**Figure 2.**
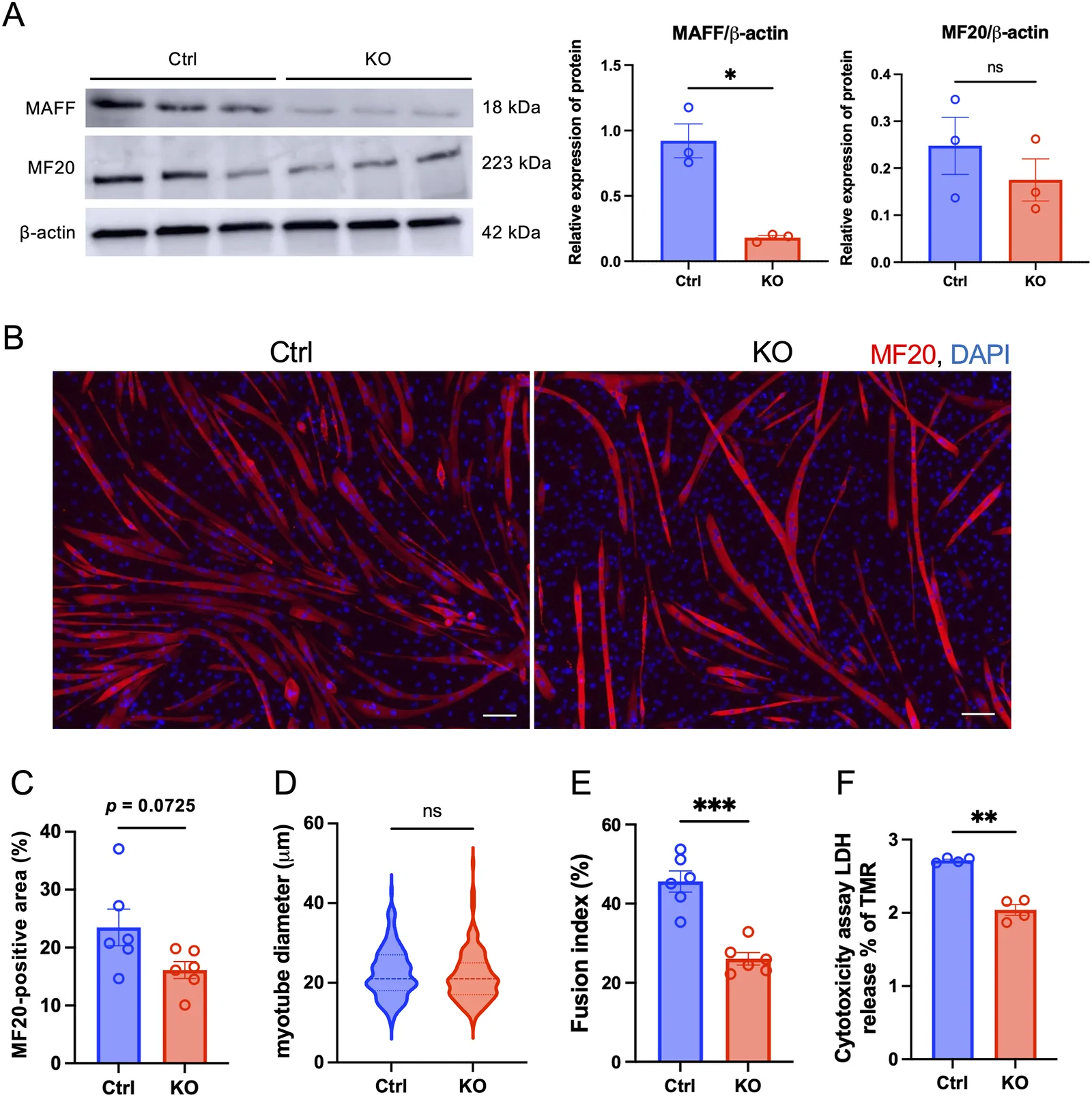
Reduced myotube fusion in *Maff* knockout (KO) C2C12 cells. **(A)** MAFF and MF20 protein levels were assessed by Western blotting in control (Ctrl) and *Maff* KO C2C12 cells at day 6 of differentiation (n = 3/group). **(B)** Representative immunofluorescence images of MF20 (red) and DAPI (blue) staining in Ctrl and *Maff* KO C2C12 myotubes at day 6 of differentiation. **(C, D)** Quantification of the MF20-positive myotube area (C; n = 6/group) and myotube diameter (D; Ctrl, n = 173; KO, n = 194) in Ctrl and *Maff* KO at day 6 of differentiation. **(E)** Fusion index analysis of Ctrl and *Maff* KO C2C12 myotubes at day 6 of differentiation (n = 6/group). **(F)** Lactate dehydrogenase (LDH) release, expressed as a percentage of the target maximum release (TMR), in Ctrl and *Maff* KO C2C12 cells (n = 4/group). In (A, C, D, E, F), data are presented as mean ± SEM. *P < 0.05; **P < 0.01; ***P < 0.001; ns, not significant. Statistical significance was determined by Welch’s *t* test.

To investigate the role of *Maff* in skeletal muscle cells, we generated *Maff* KO C2C12 myoblasts using a CRISPR/Cas9–based strategy that was previously reported (Fig S1A and B; Fukushima et al, 2024, 2026). Quantification of MAFF protein levels at day 6 after induction of differentiation confirmed a significant decrease in MAFF expression in *Maff* KO cells compared with control (Ctrl) cells (Fig 2A). To assess the impact of *Maff* deletion on myosin expression, we quantified myosin heavy chain levels using MF20. No significant difference in MF20 intensity was observed between Ctrl and *Maff* KO cells at day 6 of differentiation (Fig 2A).

**Figure S2.**
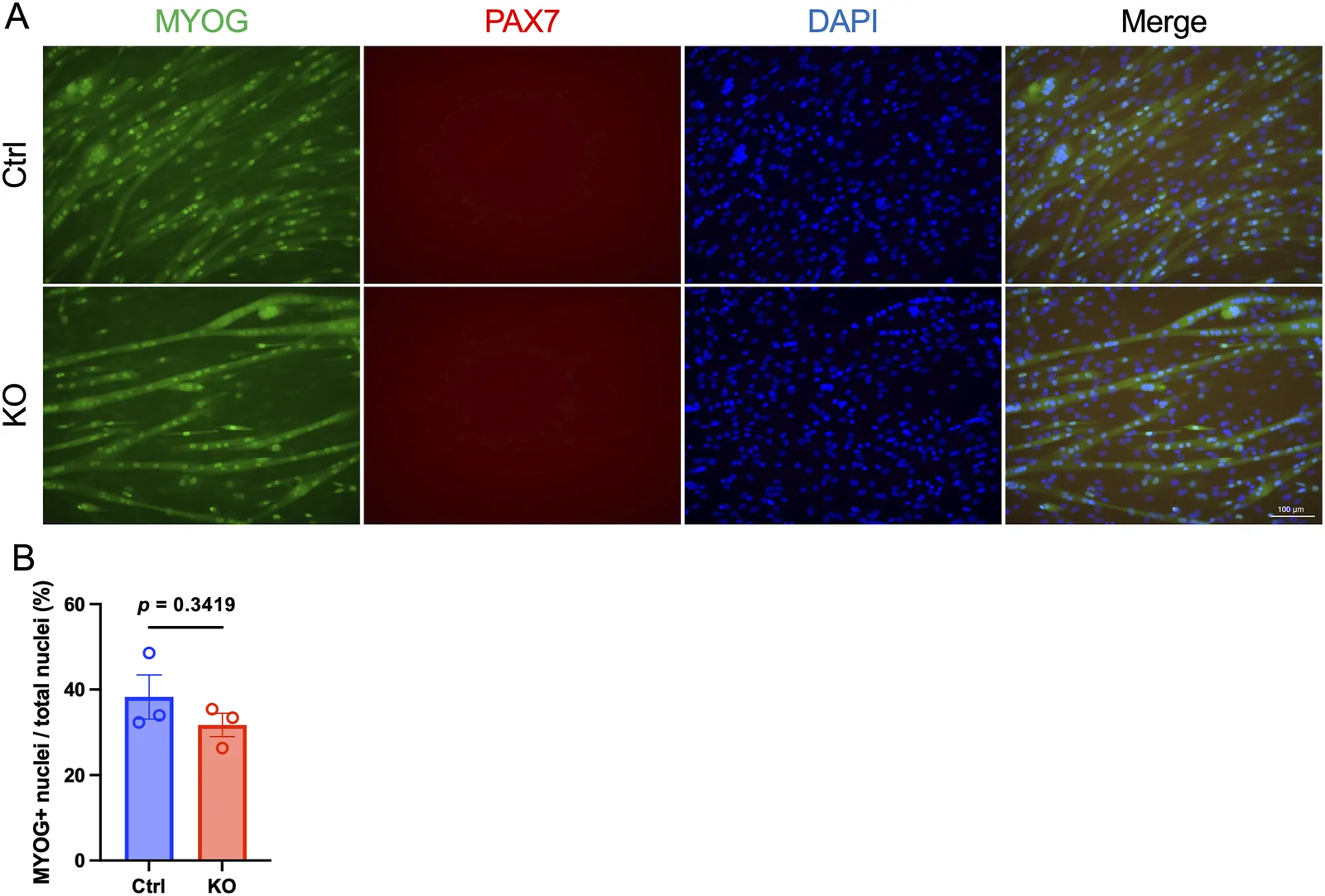
Immunofluorescence analysis of MYOG and PAX7 in Ctrl and *Maff* KO myotubes at day 7 of differentiation. **(A)** Representative immunofluorescence images of MYOG (green), PAX7 (red), and DAPI (blue) in Ctrl and *Maff* KO C2C12 myotubes at day 7 of differentiation. **(B)** Quantification of MYOG-positive nuclei relative to total DAPI-positive nuclei in Ctrl and *Maff* KO cells at day 7 of differentiation (n = 3/group). Data are presented as mean ± SEM.

Next, to examine the phenotype of *Maff* KO C2C12 myotubes, immunofluorescence analysis was performed at day 6 after differentiation (Fig 2B). Quantitative analysis revealed no significant differences between Ctrl and *Maff* KO cells in the total MF20-positive area or myotube diameter (Fig 2C and D). The fusion index (nuclei incorporated into myotubes/total number of nuclei) was significantly reduced in *Maff* KO cells compared with Ctrl cells (Fig 2E). The number of DAPI-positive nuclei was increased in *Maff* KO cells, reflecting an increased proportion of unfused mononucleated cells. To determine whether the reduced fusion index in *Maff* KO cells was associated with increased cell death, we measured lactate dehydrogenase (LDH) release and found that *Maff* KO cells showed significantly lower LDH release than Ctrl cells (Fig 2F). To further characterize the unfused mononucleated cells, we examined MYOG and PAX7 expression at day 7 of differentiation (Fig S2A). Quantification of MYOG-positive nuclei relative to total DAPI-positive nuclei showed no significant difference between Ctrl and *Maff* KO cells (Fig S2B), suggesting that the reduced fusion index was not accompanied by a detectable difference in the proportion of MYOG-positive cells. In contrast, PAX7-positive cells could not be reliably identified under our immunofluorescence conditions (Fig S2A). Therefore, we could not determine whether the increased population of unfused mononucleated cells in *Maff* KO consists of undifferentiated myogenic cells characterized by PAX7 expression. Together, these results indicate that *Maff* deficiency is associated with a reduced myotube fusion index and an increased proportion of unfused mononucleated cells, without a concomitant increase in cell death.

### *Maff* deficiency alters the transcriptional landscape of differentiated C2C12 cells

**Figure 3.**
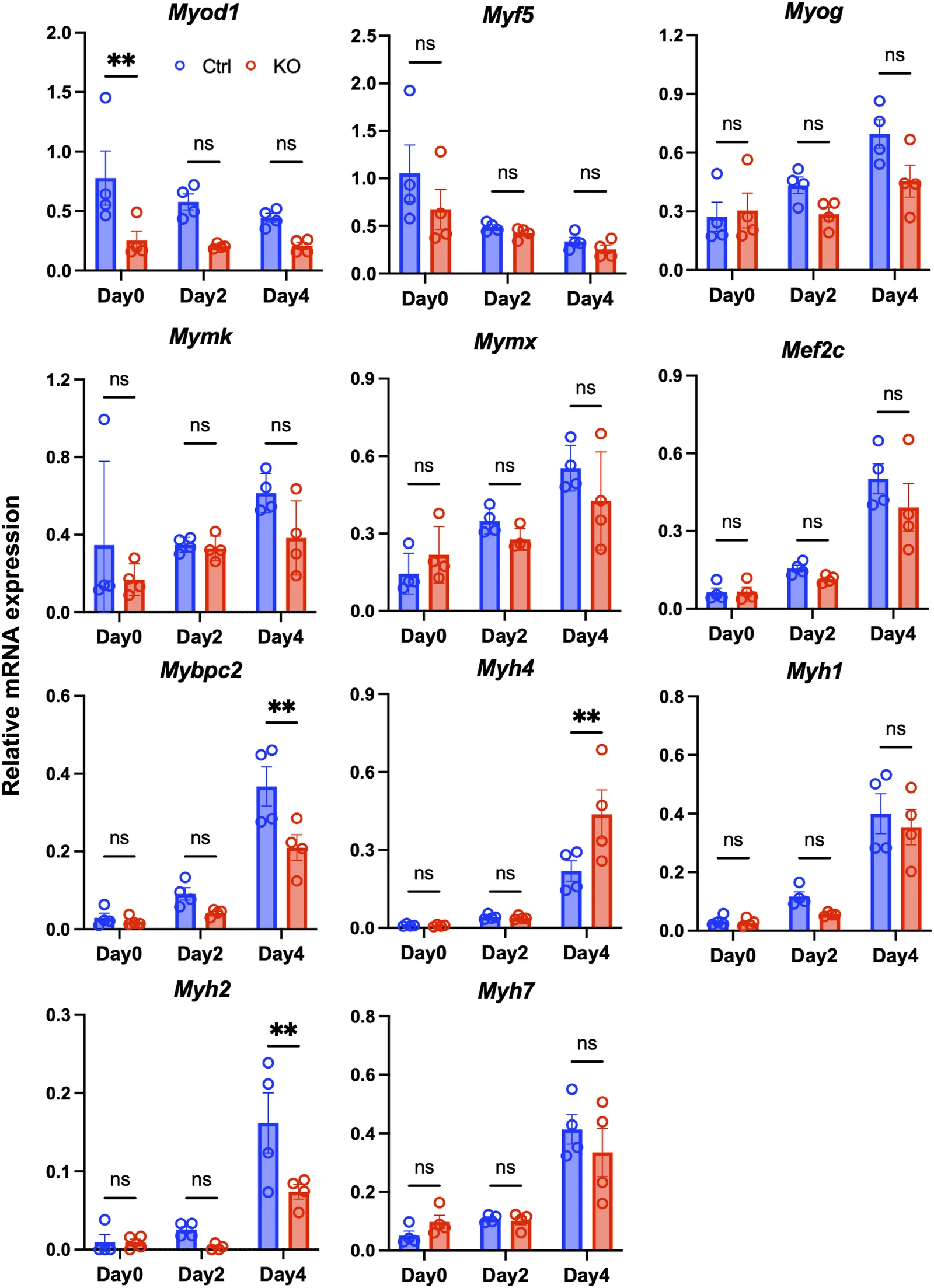
qRT-PCR analysis of myogenic markers during early differentiation. qRT-PCR analysis of myogenic differentiation and myofiber-related marker genes at days 0–4 after induction of differentiation in Ctrl and *Maff* KO cells. Data are presented as mean ± SEM. **P < 0.01; ns, not significant. Statistical significance was determined by two-way ANOVA.

To investigate the role of *Maff* during myogenic differentiation, we first analyzed the expression of myogenic differentiation and myofiber-related marker genes from day 0 to day 4 after induction of differentiation (Fig 3). Compared with Ctrl cells, expression of *Myod1* was significantly reduced in *Maff* KO cells at day 0. In addition, expression levels of *Mybpc2* and *Myh2* were significantly decreased at day 4, whereas *Myh4* expression was significantly increased at day 4 in *Maff* KO cells. By contrast, expression of the myogenic fusion-related genes *Mymk* and *Mymx* was comparable between Ctrl and *Maff* KO cells throughout differentiation. These findings suggest that the reduced fusion index observed in *Maff* KO cells is unlikely to be explained by altered expression of these canonical fusion regulators.

**Figure 4.**
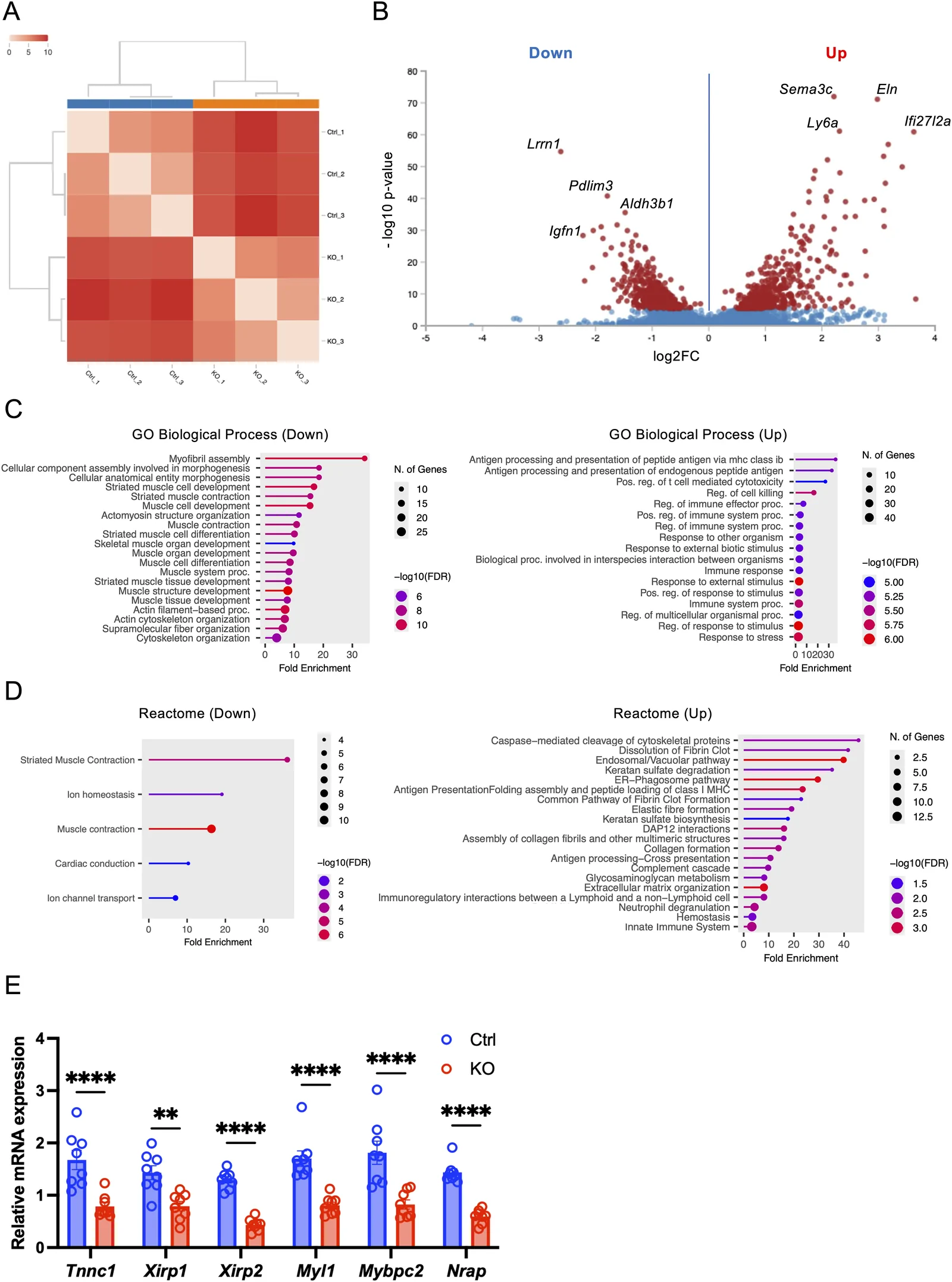
Transcriptome changes induced by *Maff* deficiency in C2C12 myotubes. **(A)** Hierarchical clustering heatmap of global gene expression profiles obtained by RNA-seq analysis in Ctrl and *Maff* KO C2C12 cells at day 7 of differentiation (n = 3/group). **(B)** Volcano plot showing the magnitude (log2 fold change) and statistical significance (−log10 *P*-value) of differentially expressed genes (DEGs) between Ctrl and *Maff* KO cells. **(C)** Gene Ontology (GO) biological process enrichment analysis of the top 100 down-regulated and up-regulated DEGs ranked by adjusted *P*-value. **(D)** Reactome pathway enrichment analysis of the top 100 down-regulated and up-regulated DEGs ranked by adjusted *P*-value. **(E)** qRT-PCR validation of selected genes involved in muscle contraction and structural organization in Ctrl and *Maff* KO C2C12 cells at day 7 of differentiation (n = 8/group). Data are presented as mean ± SEM. **P < 0.01; ****P < 0.0001. Statistical significance was determined by multiple comparisons test.

**Figure S3.**
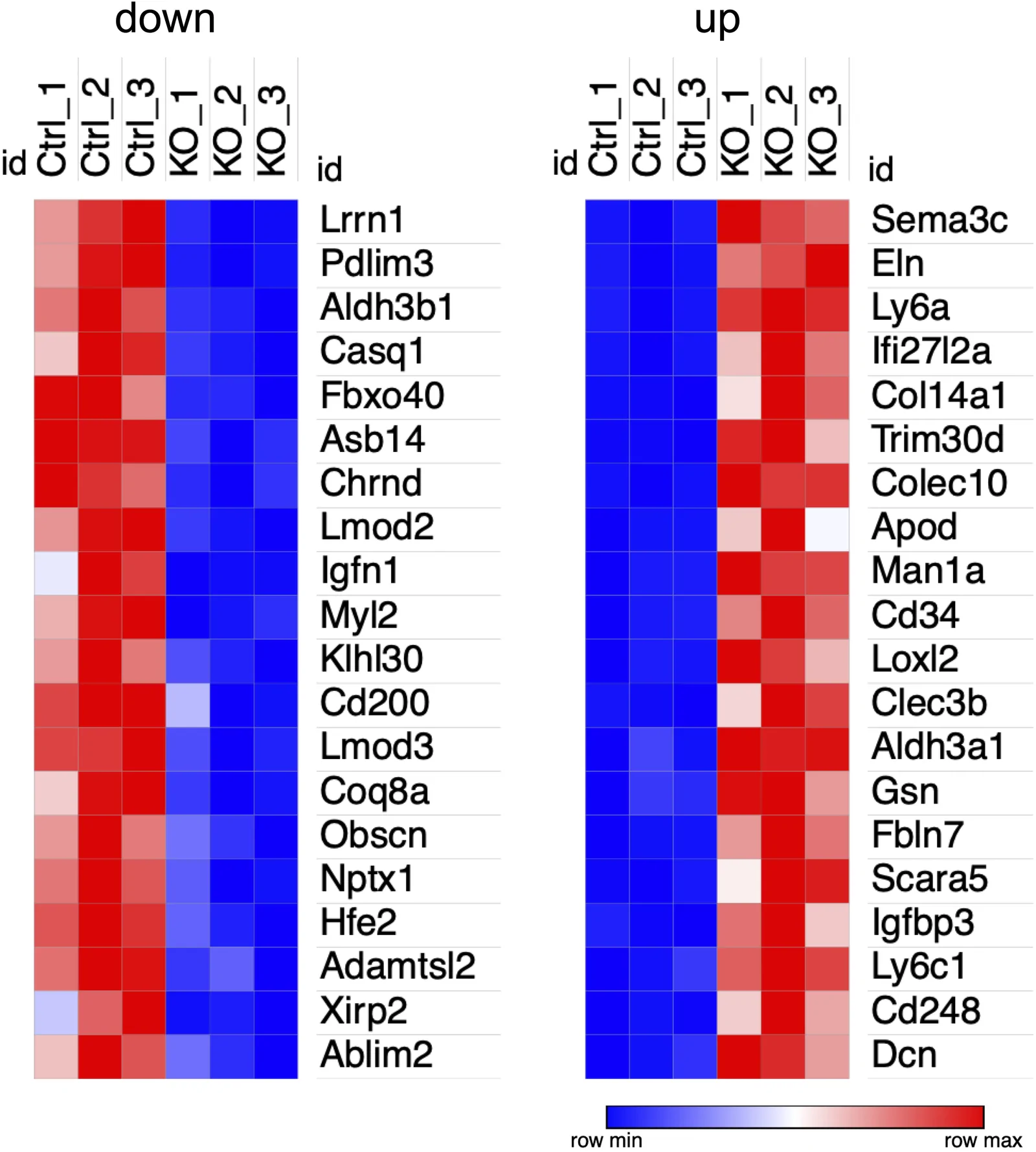
Heatmap of top 20 differentially expressed genes in *Maff* KO cells.

To comprehensively characterize transcriptional changes associated with *Maff* deficiency, we performed RNA sequencing (RNA-seq) analysis of C2C12 cells at day 7 of differentiation. Differential expression analysis identified 588 down-regulated and 687 up-regulated differentially expressed genes (DEGs) in *Maff* KO cells compared with Ctrl cells (adjusted *P* < 0.001). Among the down-regulated DEGs, 185 genes (31.46%) exhibited a log2 fold change (log2FC) of less than −1, whereas 295 genes (42.94%) of the up-regulated DEGs showed a log2FC greater than 1. Hierarchical clustering of DEGs distinguished Ctrl and *Maff* KO cells based on global gene expression patterns (Fig 4A). A volcano plot was generated to visualize the magnitude and statistical significance of differential gene expression between groups (Fig 4B). To assess the biological processes associated with *Maff*-dependent transcriptional regulation, Gene Ontology (GO) enrichment analysis was performed using the top 100 DEGs ranked by adjusted *P*-values for down-regulated and up-regulated gene sets. GO biological process analysis revealed that down-regulated DEGs were enriched for terms related to muscle differentiation, muscle contraction, and sarcomere organization, including myofibril assembly, muscle cell development/differentiation, striated muscle contraction, and actin cytoskeleton organization. In contrast, up-regulated DEGs were enriched for processes associated with immune and stress responses, such as immune response, response to external stimulus or stress, and regulation of immune system process (Fig 4C). To further identify affected molecular pathways, Reactome pathway analysis was conducted. This analysis revealed suppression of pathways related to muscle contraction and ion handling, accompanied by activation of pathways associated with immune-related processes, including caspase-dependent cytoskeletal cleavage, antigen presentation, and complement activation (Fig 4D). Consistent with these findings, heatmap analysis of the top 20 differentially expressed genes ranked by adjusted *P*-values further illustrated clear clustering of Ctrl and *Maff* KO cells (Fig S3). Notably, genes associated with muscle structure and contractile function were predominantly down-regulated, whereas genes related to extracellular matrix organization and stress responses were up-regulated in KO cells. Together with these transcriptomic and pathway analyses, quantitative real-time PCR (qRT-PCR) analysis confirmed that expression of genes involved in muscle contraction and structural organization, including *Tnnc1*, *Xirp1*, *Xirp2*, *Myl1*, *Mybpc2*, and *Nrap*, was significantly reduced in *Maff* KO cells (Fig 4E). These results indicate that loss of *Maff* is associated with widespread transcriptional reprogramming in differentiating C2C12 cells, characterized by attenuation of muscle-specific gene expression and enrichment of immune-related and extracellular matrix–associated transcriptional signatures.

### *Maff* modulates gene induction by EPS in C2C12 myotubes

**Figure 5.**
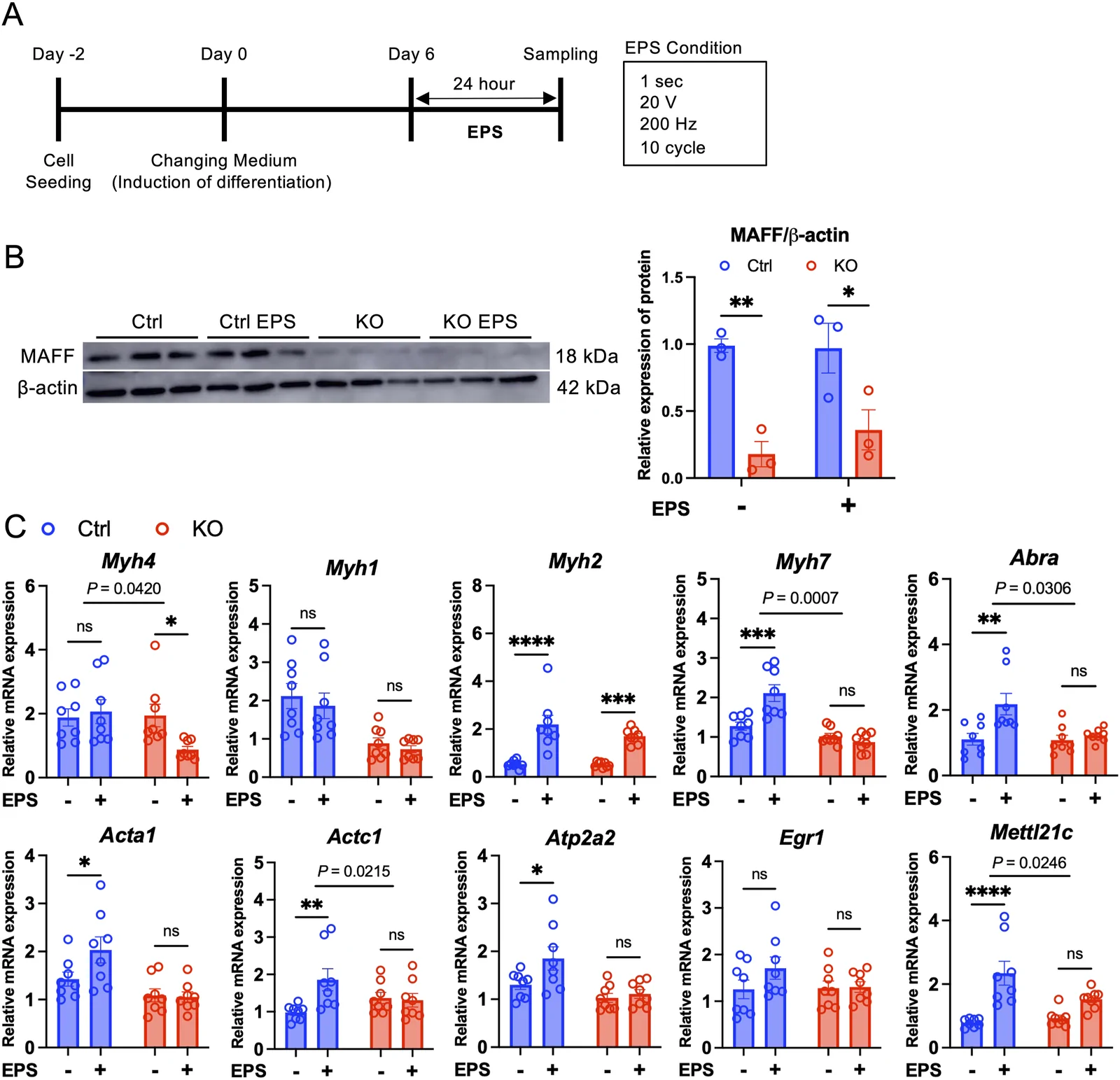
Altered EPS-induced transcriptional responses in *Maff* KO C2C12 myotubes. **(A)** Experimental scheme for EPS applied to Ctrl and *Maff* KO C2C12 cells at day 6 after induction of differentiation. **(B)** MAFF protein levels assessed by Western blotting in Ctrl and *Maff* KO C2C12 cells after EPS (n = 3/group). **(C)** qRT-PCR analysis of muscle function and myosin heavy chain-related genes in Ctrl and *Maff* KO cells after EPS. Data are presented as mean ± SEM. *P < 0.05; **P < 0.01; ***P < 0.001; ****P < 0.0001; ns, not significant. Statistical significance was determined by Welch’s *t* test or two-way ANOVA.

**Figure S4.**
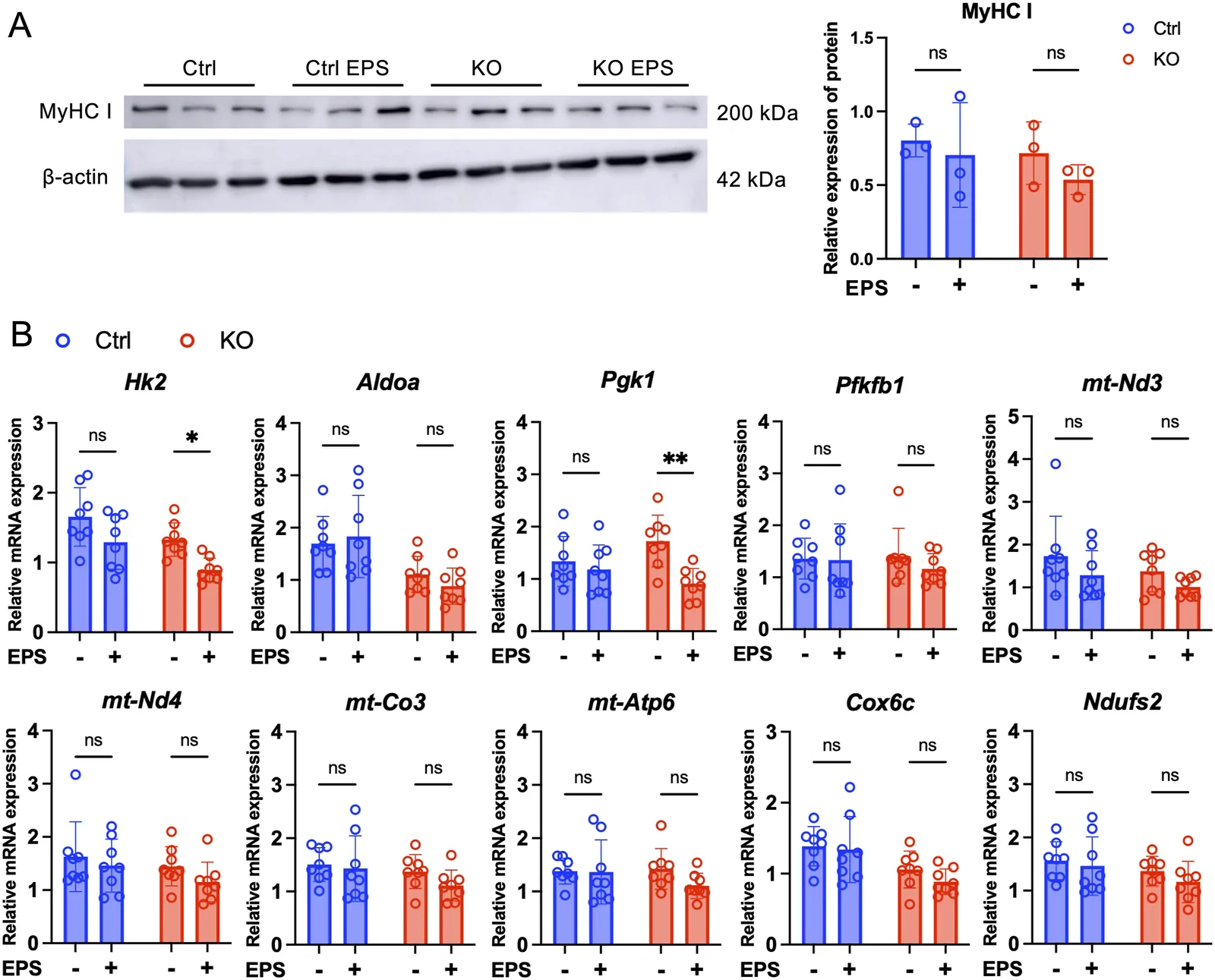
Effects of MAFF deficiency on muscle and metabolic responses to EPS. **(A)** MyHC type I protein levels assessed by Western blotting in Ctrl and *Maff* KO C2C12 myotubes after EPS (n = 3/group). **(B)** qRT-PCR analysis of glycolytic and mitochondrial gene expression in Ctrl and *Maff* KO C2C12 myotubes after EPS. Data are presented as mean ± SEM. *P < 0.05; **P < 0.01; ns, not significant. Statistical significance was determined using Welch’s *t* test for (A) and two-way ANOVA for (B).

We observed that *Maff* expression is induced by EPS in wild-type C2C12 myotubes (Fig 1D). To investigate whether *Maff* contributes to EPS-induced transcriptional responses, EPS was applied to *Maff* KO cells at day 6 after induction of differentiation (Fig 5A). We observed visible contractions in both Ctrl and *Maff* KO myotubes during EPS stimulation (Video 1 and Video 2), confirming that both cell types responded to the stimulation. Western blot analysis confirmed that MAFF protein expression was significantly reduced in *Maff* KO cells compared with Ctrl cells under both basal and EPS conditions (Fig 5B). EPS did not significantly alter MAFF protein abundance, but *Maff* KO cells consistently exhibited significantly lower MAFF levels than Ctrl cells. We also examined the slow-twitch fiber marker MyHC I and found no significant differences in its protein levels between Ctrl and *Maff* KO cells under either basal or EPS conditions (Fig S4A). We next examined the expression of muscle function and fiber-type specification genes by qRT-PCR (Fig 5C). In Ctrl cells, EPS significantly increased the expression of several genes involved in muscle contraction, calcium handling, and adaptive remodeling, such as *Myh2*, *Myh7*, *Abra*, *Acta1*, *Actc1*, *Atp2a2*, and *Mettl21c*. In contrast, these EPS-induced transcriptional responses were not significant in *Maff* KO cells. Notably, the induction of *Myh7* and *Mettl21c*, which are known to be associated with protein degradation or slow-twitch muscle programs (Wiederstein et al, 2018), were induced by EPS stimulation in Ctrl cells, whereas this induction was attenuated in KO cells. Similarly, EPS-dependent increases in *Abra*, *Acta1*, *Actc1*, and *Atp2a2* were observed in Ctrl cells but were significantly blunted or lost in KO cells. By contrast, the expression of *Myh1* and *Egr1* showed no significant response to EPS in either cell type. Furthermore, *Myh4* expression was reduced by EPS in KO cells but not in Ctrl cells. To further assess metabolic gene expression, we examined representative glycolytic genes (*Hk2*, *Aldoa*, *Pgk1*, and *Pfkfb1*) and mitochondrial-related genes (*Nd3*, *Nd4*, *Co3*, *Atp6*, *Cox6c*, and *Ndufs2*) by qRT-PCR. Overall, no consistent differences in the expression of these genes were observed between Ctrl and *Maff* KO cells after EPS stimulation (Fig S4B), suggesting that MAFF deficiency has minimal effects on these metabolic gene programs under EPS conditions. Together, these results indicate that MAFF is required for the proper transcriptional activation of multiple EPS-responsive genes in C2C12 myotubes, suggesting that MAFF contributes to the regulation of contraction-induced gene programs in myotubes.

Video 1 Representative video of EPS-induced contraction in Ctrl C2C12 myotubes. Download video

Video 2 Representative video of EPS-induced contraction in *Maff* KO C2C12 myotubes. Download video

## Discussion

In this study, we examined the potential contribution of the small *Maf* family member MAFF to skeletal muscle–related transcriptional programs by integrating public exercise transcriptomic datasets, in vivo exercise experiments, and C2C12-based in vitro stimulation models. Across human and mouse datasets, *Maff* expression increased after acute exercise, and we observed consistent induction in exercised mouse muscles and in electrically stimulated C2C12 myotubes (Fig 1). Genetic ablation of *Maff* in C2C12 cells was associated with reduced myogenic fusion without changes in myosin heavy chain or myotube size, together with broad transcriptomic changes marked by decreased expression of muscle structural and contractile genes, and enrichment of immune-, interferon-, and extracellular matrix-associated signatures (Figs 2 and 4). In addition, *Maff* deficiency attenuated EPS-induced gene expression responses, such as *Abra*, *Acta1*, *Actc1*, *Atp2a2*, *Myh7*, and *Mettl21c* (Fig 5). Collectively, these results link MAFF to exercise and contraction-induced transcriptional programs in skeletal muscle cells and associate MAFF loss with impaired myogenic fusion and altered stimulus responsiveness.

Our transcriptomic analyses further indicate that *Maff* deficiency is associated with a shift away from mature myogenic gene expression and toward signatures linked to immune/stress responses and extracellular matrix remodeling. In other organs, MAFF expression is inducible by proinflammatory cytokines and MAFF has been implicated in inflammation-linked transcriptional networks in vivo. Moreover, the murine *Maff* locus has been molecularly characterized with respect to promoter usage and tissue expression patterns (Onodera et al, 1999; Massrieh et al, 2006; von Scheidt et al, 2021). In this context, the immune/interferon-associated and extracellular matrix–associated signatures observed in *Maff*-deficient myotubes may reflect direct MAFF-dependent regulation, secondary consequences of impaired myogenic fusion and incomplete maturation, or a combination of both. The established role of sMafs (MafF, MafG, and MafK) in antioxidant response element (ARE)-associated cytoprotective programs and stress adaptation in vivo (Yamazaki et al, 2012) is consistent with the possibility that loss of MAFF alters how differentiating myotubes accommodate cellular stress or redox-related cues during maturation and stimulation, thereby reshaping downstream transcriptional programs. Clarifying whether immune/interferon-associated gene induction represents a primary transcriptional consequence of MAFF loss or a secondary feature of defective differentiation will require mapping MAFF-centered regulatory complexes and their genomic targets at higher resolution.

In the present study, we used EPS as an in vitro model of contractile activity and performed transcriptome-wide analyses in *Maff* KO C2C12 myotubes. Genes differentially responsive to stimulation between Ctrl and *Maff* KO cells were enriched for sarcomere-related transcripts, including *Myh7*, *Actc1*, and *Abra*, indicating that MAFF modulates a subset of contraction-responsive gene programs. Notably, *Maff* itself was induced by EPS in wild-type myotubes, suggesting that MAFF responds to mechanical or contractile cues and contributes to shaping downstream transcriptional responses. A recent study demonstrated that the large MAF family member c-MAF is required to translate motor neuron-derived activity into transcriptional programs that maintain fast-glycolytic muscle characteristics and neuromuscular junction integrity (Jauliac et al, 2026 *Preprint*). Although MAFF belongs to the sMAF subfamily and lacks an intrinsic transcriptional activation domain, our findings raise the possibility that sMAF proteins also participate in activity-dependent gene regulation in skeletal muscle. Because small MAF proteins exert their transcriptional functions through homo- or heterodimerization, the absence of detectable changes in MAFF protein abundance after EPS suggests that its activity may instead be regulated by mechanisms such as posttranslational modification, subcellular localization, or interactions with partner proteins (Jauliac et al, 2026 *Preprint*). sMafs are known to bind T-MARE (TGCTGACTCAGCA) and C-MARE (TGCTGACGTCAGCA) motifs (Kataoka et al, 1995; Katsuoka & Yamamoto, 2016) and function through heterodimerization with bZIP partners such as CNC and Bach family proteins, raising the possibility that EPS-induced MAFF regulates stimulation-responsive genes via MARE-containing regulatory elements. However, direct genomic targets were not defined in this study, and chromatin-based approaches will be required to establish MAFF-dependent regulatory mechanisms.

Finally, although EPS captures key aspects of contraction-induced signaling, it does not fully reproduce the systemic and temporal complexity of in vivo exercise (Nikolić et al, 2017; Carter & Solomon, 2019). Given that *MAFF* is induced after short-term but not long-term exercise in available datasets (Pillon et al, 2020), the 24-h EPS paradigm used here may not directly correspond to specific exercise modalities. This limitation should be considered in interpreting these findings and addressed in future studies using diverse stimulation paradigms. Moreover, only a subset of myotubes exhibited visible contractions in the representative EPS recordings. Thus, the proportion of contracting myotubes may have been insufficient to produce detectable changes in MAFF expression using bulk qRT-PCR or Western blot analyses. Based on the data presented in Figs 2 and 3, we also cannot exclude the possibility that myogenic differentiation is delayed in *Maff* KO cells, which may have contributed to the observed effects in the EPS experiment. Furthermore, whereas the in vivo analyses were performed in mature skeletal muscle, the *Maff* KO experiments in this study were conducted in muscle progenitor cells before differentiation. Therefore, it remains unclear to what extent the observed phenotypes reflect the role of MAFF in fully differentiated myotubes. Future studies using inducible knockdown or knockout approaches in differentiated myotubes will help clarify the specific role of MAFF in mature muscle cells. In addition, because the *Maff* KO cells used in this study were generated as a polyclonal population rather than isolated single clones, the presence of residual WT cells cannot be excluded. Consequently, these cells may partially contribute to the observed myotube phenotypes and attenuate the effects of MAFF deficiency.

## Materials and Methods

### Mouse treadmill exercise and tissue collection

8-wk-old C57BL/6J male mice (Japan SLC, Inc.) were used. For treadmill exercise experiments, male mice ran at 15 m/min for 30 min on a 5° incline on the treadmill (MK-680AT/02M; Muromachikikai). Sedentary control mice were maintained without exercise. Gastrocnemius (GA) and soleus (Sol) muscles were collected 30 min after completion of exercise. The mice were euthanized by cervical dislocation. The animal experiment in this study was approved by the Animal Research Committee of Aichi Medical University (Permission number: 2282).

### Cell culture

C2C12 cells were kindly provided by Dr. Shinichiro Hayashi. Cells were maintained in high-glucose Dulbecco’s Modified Eagle’s Medium (DMEM; 4.5 g/liters glucose; D6429-500ML; Sigma-Aldrich) supplemented with 10% fetal bovine serum (FBS; 10099-141; Thermo Fisher Scientific) and 1% penicillin–streptomycin (P/S; 26253-84; Nacalai Tesque) at 37°C in 5% CO_2_. For differentiation experiments, C2C12 myoblasts were grown to 80% confluence and seeded at 2 × 10^5^ cells per well in 8-well plates (167064; Thermo Fisher Scientific). Myogenic differentiation was induced 2 d after seeding by switching to DMEM containing 2% horse serum (HS; 16050-122; Thermo Fisher Scientific) and 1% P/S. Differentiation medium was replaced every 48 h.

At day 6 after induction of differentiation, differentiated myotubes were used for electrical pulse stimulation (EPS) experiments or clenbuterol. For clenbuterol treatment, the medium was replaced with low-glucose DMEM (1 g/liters glucose; D6046-500ML; Sigma-Aldrich) supplemented with 2% HS, 1% P/S, and 30 ng/ml clenbuterol (032-20441; FUJIFILM Wako Pure Chemical).

### Immunofluorescence staining

C2C12 myotubes at day 6 of differentiation were fixed with 4% paraformaldehyde–phosphate buffer in phosphate-buffered saline (PBS; 09154-85; Nacalai Tesque) for 10 min, and then permeabilized with PBS containing 0.1% Triton at room temperature. The cells were washed three times with PBS (5 min each) and blocked in PBS containing 0.1% Triton and 2% HS for 30 min at room temperature. Primary antibodies were diluted in blocking buffer and applied as follows: anti-Myosin Heavy Chain (MyHC) antibody (MF20 clone, MAB4470; R&D Systems, 1:100), anti-MYOG (sc-576; Santa Cruz Biotechnology, 1:100), and anti-PAX7 (sc-81648; Santa Cruz Biotechnology, 1:100). Samples were incubated overnight at 4°C. After washing with PBS, the cells were incubated with Alexa Flour 555–conjugated anti-mouse IgG (A28180; Thermo Fisher Scientific, 1:1,000) for the detection of MF20 and PAX7, and Alexa Flour 488–conjugated anti-rabbit IgG (A11008; Life technologies, 1:1,000) for the detection of MYOG for 1 h at room temperature in the dark. Nuclei were counterstained with DAPI (340-07971; FUJIFILM Wako Pure Chemical). Images were acquired using a fluorescence microscope (BZ-X800; KEYENCE) with a 10× objective and quantified with ImageJ software.

### Plasmid construction

Distinct guide RNAs (gRNAs) targeting the mouse *Maff* gene were designed using the CHOPCHOP web tool (Labun et al, 2019). All oligonucleotides were purchased from Fasmac Co., Ltd. Annealed oligonucleotide pairs encoding each gRNA (gRNA1-gRNA4) or a negative control (NC) gRNA were cloned into the pGuide-it-ZsGreen vector (Z2601N; TaKaRa) according to the manufacturer’s instructions. The resulting plasmids were designated pGuide-it-ZsGreen-*Maff*_1, pGuide-it-ZsGreen-*Maff*_2, pGuide-it-ZsGreen-*Maff*_3, pGuide-it-ZsGreen-*Maff*_4, and pGuide-it-ZsGreen-NC. Sequences are provided in Table S1. All plasmid sequences were verified by sequencing.

Table S1. The sequences of the inserted oligonucleotides for *Maff* KO cell.

### Generation of *Maff* KO C2C12 cells

The overall workflow for generating KO cells was described previously (Fukushima et al, 2024). To generate *Maff*-deficient C2C12 cells, four plasmids (pGuide-it-ZsGreen- *Maff*_1, pGuide-it-ZsGreen- *Maff*_2, pGuide-it-ZsGreen- *Maff*_3, and pGuide-it-ZsGreen- *Maff*_4) were pooled and co-transfected into C2C12 myoblasts. As a control (Ctrl), C2C12 cells were transfected with pGuide-it-ZsGreen-NC. Transfections were performed using jetPRIME reagent (101000027; Polyplus) according to the manufacturer’s instructions. Briefly, C2C12 cells were seeded at 1 × 10^6^ cells per well in 100 mm plates and cultured in DMEM supplemented with 10% FBS for 24 h before transfection. A pooled plasmid mixture (500 ng of each gRNA plasmid) or the control plasmid was then transfected using jetPRIME.

At 24 h after transfection, ZsGreen fluorescence was confirmed by fluorescence microscopy. ZsGreen-positive cells were isolated by fluorescence-activated cell sorting using a cell sorter (FACSAria III, Waters Biosciences). Sorted cells were expanded for 6 d and subsequently cryopreserved at −80°C.

### Western blot analysis

C2C12 myotubes at day 6 of differentiation were homogenized in lysis buffer and clarified by centrifugation. Protein concentrations were determined using a Protein Assay BCA Kit (297-73101; FUJIFILM Wako Pure Chemical). A total of 10 μg protein per sample was separated on 4–15% Mimi-PROTEAN TGX Precast Protein Gels (4561086; Bio-Rad) and transferred to PVDF membranes. Membranes were blocked with 3% milk in TBST and probed with primary antibodies against MAFF (12771-1-AP; Proteintech, 1:1,000), Myosin Heavy Chain (MyHC) antibody (MF20 clone, MAB4470; R&D Systems, 1:1,000), MyHC I (M8421; Sigma-Aldrich, 1:1,000), and β-Actin (010-27841; FUJIFILM Wako Pure Chemical, 1:1,000), followed by anti-rabbit IgG-HRP (SA00001-2; Proteintech, 1:10,000) for MAFF detection and anti-mouse IgG-HRP (SA00001-1; Proteintech, 1:10,000) for MF20, MyHC I, and β-Actin detection. Signals were detected using an ImageQuant LAS 500 (Cytiva) and quantified with ImageJ software.

### RNA extraction

Cryopreserved Ctrl and *Maff* KO cells were differentiated into myotubes. Total RNA was extracted at day 0, 2, and 4 after induction of differentiation, as well as after 24 h of EPS stimulation, using RNAiso Plus Reagent (9109; TaKaRa) according to the manufacturer’s instructions. RNA concentration was measured with a NanoDrop 1000 Spectrophotometer (Thermo Fisher Scientific), and RNA purity was assessed by the 260 nm/280 nm absorbance ratio. The extracted RNA samples were stored at −80°C.

### Quantitative real-time PCR

Total RNA (500 ng) was reverse-transcribed using PrimeScript RT Master Mix (RR036A; TaKaRa). Quantitative real-time PCR (qRT-PCR) was performed in a final volume of 10 μl using GeneAce SYBR qPCR Mix II (313-09423; NIPPON GENE) according to the manufacturer’s instructions on a StepOne Real-Time PCR System (Applied Biosystems). Primer sequences are listed in Table S2. Relative mRNA expression levels were normalized to 18S ribosomal RNA (*Rn18s*) and quantified using the standard curve method, except for the exercise experiments shown in Fig 1, in which *Actb* was used as the internal control.

Table S2. The sequences of the oligonucleotides for quantitative RT-PCR.

### Electrical pulse stimulation

EPS was performed using a C-Dish system (CLD8WN; IonOptix) connected to a function generator (FGX-2220; Texio) and an amplifier (T-HVA03; Turtle Industry). For EPS, the medium was changed to high-glucose DMEM (4.5 g/liters glucose) containing 2% HS and 1% P/S. Cells were stimulated for 24 h with electrical pulses (20 V, 200 Hz, 5-ms pulse width) delivered as 1-s burst trains repeated for 10 cycles. EPS-induced muscle contraction was confirmed using an optical microscope (IMT-2; Olympus) and a microscope camera adapter (3R-DKMC01; 3R Systems) with a 10× objective. After stimulation, the cells were lysed with RNAiso Plus Reagent (9109; TaKaRa) and stored in a freezer at −80°C.

### RNA-seq and bioinformatics analysis

Total RNA extracted from C2C12 cells at day 7 after induction of differentiation was used for RNA-seq. Libraries were prepared using NEBNext Poly(A) mRNA Magnetic Isolation Module NEBNext Ultra II Directional RNA Library Prep Kit (New England Biolabs) according to the manufacturer’s instructions. Sequencing was performed on an Illumina NovaSeq X Plus (Illumina) using 150-bp paired-end reads (PE150), yielding approximately 40 million reads per sample. Sequenced reads were processed using the RaNA-seq framework and mapped to the mouse reference genome (GRCm38). Differential expression analysis was performed using DESeq2. Hierarchical clustering analysis of the data was performed as previously described (Prieto & Barrios, 2020). RNA-seq data were deposited in the Gene Expression Omnibus (accession GSE324257). GO biological process and Reactome pathway enrichment analyses were performed using ShinyGO (v0.81) (Xijin Ge et al, 2020). Hierarchically clustered heatmaps were generated using Morpheus software (https://software.broadinstitute.org/morpheus).

### LDH release assay

Cytotoxicity was assessed by measuring lactate dehydrogenase (LDH) release using the Cytotoxicity LDH Assay Kit-WST (Dojindo) according to the manufacturer’s protocol. Briefly, the culture supernatants were transferred to a 96-well plate (92696; TPP), and LDH activity was determined after addition of the kit working solution. To generate a high-LDH control, the cells were lysed using the kit lysis buffer and incubated at 37°C for 30 min. After color development, the reaction was stopped using the kit stop solution, and absorbance was measured at 490 nm using a SpectraMax ABS Plus microplate reader (Molecular Devices).

### Statistical analysis

Results are expressed as mean ± standard error of the mean (SEM). Comparisons between two groups were performed using unpaired Welch’s *t* tests. For comparisons among multiple groups, multiple comparisons test and two-way analysis of variance (ANOVA) followed by Tukey’s honestly significant difference (HSD) post hoc test were used. Statistical significance is denoted as follows: *P < 0.05; **P < 0.01; ***P < 0.001; ****P < 0.0001.

## Supplementary Material

Reviewer comments

## Data Availability

All RNA-seq files are available from the NCBI database (accession number GSE324257).
